# Identification of *Peptoniphilus vaginalis*-Like Bacteria, *Peptoniphilus septimus* sp. nov., From Blood Cultures in a Cervical Cancer Patient Receiving Chemotherapy: Case and Implications

**DOI:** 10.3389/fcimb.2022.954355

**Published:** 2022-07-08

**Authors:** Huacheng Wang, Jin-Lei Yang, Chunmei Chen, Ying Zheng, Mingming Chen, Junhua Qi, Shihuan Tang, Xiao-Yong Zhan

**Affiliations:** ^1^ The Seventh Affiliated Hospital, Sun Yat-sen University, Shenzhen, China; ^2^ School of Clinical Medicine, Guangdong Pharmaceutical University, Guangzhou, China

**Keywords:** *Peptoniphilus*, *Peptoniphilus septimus*, *Peptoniphilus vaginalis*, cervical cancer, bloodstream infection, MALDI–TOF, whole-genome sequencing, genome-based taxonomy

## Abstract

A 39-year-old woman with a 3-year human papillomavirus (HPV) 18 infection history was admitted to the hospital for a 16-day history of vaginal bleeding after sex. She was diagnosed with cervical cancer based on the results of the electronic colposcopy, cervical cytology, microscopy, and magnetic resonance imaging (MRI). Then, she received chemotherapy, with paclitaxel 200 mg (day 1), cisplatin 75 mg (day 2), and bevacizumab 700 mg (day 3) twice with an interval of 27 days. During the examination for the diagnosis and treatment, many invasive operations, including removal of intrauterine device, colposcopy, and ureteral dilatation, were done. After that, the patient was discharged and entered the emergency department about 2.5 months later with a loss of consciousness probably caused by septic shock. The patient finally died of multiple organ failure and bacterial infection, although she has received antimicrobial therapy. The blood cultures showed a monobacterial infection with an anaerobic Gram-positive bacterial strain, designated as SAHP1. Matrix-assisted laser desorption ionization time-of-flight mass spectrometry (MALDI–TOF MS) indicated that the patient was infected with *Peptoniphilus asaccharolyticus*, while molecular analysis and genome-based taxonomy confirmed the infection with a novel *Peptoniphilus* species that has a close genetic relationship with *Peptoniphilus vaginalis* and proposed provisionally as *Peptoniphilus septimus* sp. nov., which may also act as a commensal of the human vagina. Genomic features of SAHP1 have been fully described, and comparative genomic analysis reveals the known prokaryote relative of *Peptoniphilus septimus* sp. nov. in the genus *Peptoniphilus.* The invasive operations on the genital tract during the diagnosis and treatment of the patient and the tumor tissue damage and bleeding may have a certain role in the bloodstream infection. This study casts a new light on the *Peptoniphilus* bacteria and prompts clinicians to include anaerobic blood cultures as part of their blood culture procedures, especially on patients with genital tract tumors. Furthermore, due to the incomplete database and unsatisfying resolution of the MALDI–TOF MS for *Peptoniphilus* species identification, molecular identification, especially whole-genome sequencing, is required for those initially identified as bacteria belonging to *Peptoniphilus* in the clinical laboratory.

## Introduction

The genus *Peptoniphilus* is a group of Gram-positive anaerobic coccus (GPAC), which always acts as normal microbiota that mostly colonizes the mucosal surfaces of the mouth and the gastrointestinal and genitourinary tracts (Murphy and Frick, 2013). It could also be a pathogen for human diseases because of its isolation from various clinical specimens including vaginal discharges and ovarian, peritoneal, and gland abscesses (Murdoch, 1998; Mishra et al., 2012). *Peptoniphilus* species, including *P. vaginalis*, have also reportedly been frequently isolated from vaginal discharges and associated with bacterial vaginosis, indicating that they have a close relationship with the genital tract (Sharma et al., 2014; Petrina et al., 2017). They can also be isolated from ovarian abscesses, retroperitoneal abscesses, and spinal fluid, showing certain pathogenicity in some cases (Puapermpoonsiri et al., 1996; Brook, 1999; Ezaki et al., 2001; Okui et al., 2016; Diop et al., 2016; Diop et al., 2019). Members of *Peptoniphilus* can also cause many severe infections in the peritoneum, osteoarticular skin, lymphocele, breast, bone and joint, soft tissue, surgical site infections, and blood (Fenollar et al., 2006; Dowd et al., 2008; Murphy and Frick, 2013; Seng et al., 2015; Cobo et al., 2017; Verma et al., 2017; Cobo, 2018; Le Bihan et al., 2019; Muller-Schulte et al., 2019; Wan et al., 2021). However, to date, no case of *Peptoniphilus* infection has been reported in patients with genital tract tumors, although many of these bacteria colonize the genitourinary tract (Murphy and Frick, 2013; Diop et al., 2016; Diop et al., 2019). Herein, we report a case of bloodstream monoinfection of *P. vaginalis*-like bacteria in a cervical cancer patient receiving chemotherapy. The molecular identification of the strain indicated that it is a novel *Peptoniphilus* sp. that has a close genetic relationship with *P. vaginalis* and *P. harei*, with *P. vaginalis* the closest, designated as *P. septimus*. Our findings highlight that the prevalence of *Peptoniphilus* bacteria and species in the clinic may have been underestimated. More awareness should be paid regarding the pathogenic potential of these opportunistic bacteria in the genus *Peptoniphilus*. In addition, *P. septimus* may be misidentified as *P. asaccharolyticus* in the clinical laboratory when using the VITEK MS matrix-assisted laser desorption ionization time-of-flight mass spectrometry (MALDI–TOF MS) probably because the two species have similar biochemical characteristics and are difficultly differentiated by proteome phenotyping such as that found between *P. harei* and *P. asaccharolyticus* (Wan et al., 2021), raising the importance of molecular identity, especially the whole-genome sequencing (WGS) for genus *Peptoniphilus* bacteria.

## Materials and Methods

### Ethics Statement

This work was approved by the Ethics Committee of the Seventh Affiliated Hospital, Sun Yat-sen University. No personal identification data or potentially identifiable images were included in this article.

### Case Description

**Figure 1:**
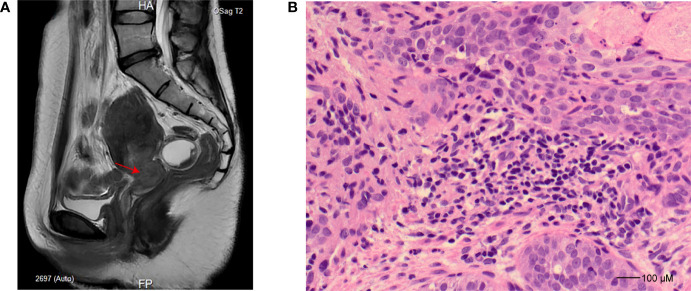
Imaging and histopathology study for the patient. **(A)** Sagittal T2-weighted turbo spin-echo sequence, slightly hyperintense tumor in the cervix (indicated as an arrow). **(B)** Hematoxylin and eosin staining (×40) for cervical biopsy at the time of cervical cancer diagnosis demonstrates poorly differentiated squamous cell carcinoma characterized by pleomorphism and high nuclear-to-cytoplasmic ratios.

A 39-year-old woman was admitted to the hospital for a 16-day history of vaginal bleeding after sex and a 3-year human papillomavirus (HPV) 18 infection on September 6, 2021. The patient has an intrauterine device (IUD) put into the uterus in 2013. For further magnetic resonance imaging (MRI) tests, she has undergone IUD removal and curettage 1 day after admission. No abnormal signs were observed during and after the IUD removal and curettage. Electronic colposcopy and cervical cytology indicated HPV 18 infection and showed that the cervix was withered and small, and the vaginal fornix disappeared. Gray cauliflower polypoid–like vegetation with a diameter of 1 cm was seen at the mouth of the cervix. MRI showed a space-occupying lesion of the cervix (**Figure 1A**). Cervical biopsy specimens showed heterotypic cell-in-cell structures in the tissue, with large and deeply stained nuclei, which were consistent with the cell morphology of poorly differentiated cervical cancer (**Figure 1B**). Immunohistochemistry showed that the cancer cells have strong and diffuse p16 staining, the proportion of Ki67-positive cells was about 60%, and the cells were CK5/6 positive, p63 positive, P40 positive, and CK8/18 positive but ab-pas negative. The patient was finally diagnosed with cervical cancer and planned to receive radiotherapy and chemotherapy. MRI and computed tomography (CT) also showed tumor invasion of the left ureter and effusion of the left ureter and kidney. Thus, she received ureteral dilatation and stent insertion 11 days after admission. Rehydration, antimicrobial, pain relief, and other symptomatic treatment and supportive care were applied to the patient after this operation. The patient’s vital signs were stable after the operation, without special discomfort, low back pain, nausea, vomiting, chills, fever, etc. On September 18, 2021, the patient started to receive chemotherapy treatments with paclitaxel 200 mg (day 1), cisplatin 75 mg (day 2), and bevacizumab 700 mg (day 3). Liver injury after chemotherapy was observed, manifesting as elevated alanine aminotransferase and aspartate aminotransferase (48 U/L and 53 U/L on September 22, 2021, and 85 U/L and 72 U/L on September 23, 2021, respectively; reference normal values are both <40 U/L). After symptomatic treatment, the patient was self-discharged.

On October 11, 2021, the patient was admitted again with a chief complaint of chronic lower abdominal pain and received the second round of chemotherapy treatments during October 14 and October 16. The drug dosage was the same as the first round. The patient was self-discharged on October 18, 2021. The patient experienced a half-month of abdominal pain and diarrhea before the last admission. On December 29, the patient experienced a sudden loss of consciousness without convulsions and foaming at the mouth but with fecal incontinence at 18:31 and entered the emergency department of the hospital. Physical examination showed the disappearance of carotid pulse and weakness of breathing. After cardiopulmonary resuscitation, the heart rate of the patient recovered to about 40 beats/min. Atropine and amiodarone were administered to control the ventricular rate, but cardiac arrest occurred again at 18:46. After a second-round cardiopulmonary resuscitation, the patient’s heart was back into a normal rhythm at 18:55. Detailed physical examination showed pulse 130/min (reference normal range is 60–100/min), respirations 45/min (reference normal range is 12–16/min), arterial oxygen saturation (SPO2) 96% (reference normal range is ≥95%), and blood pressure 113/68 mmHg (reference normal range is 90–120/60–90 mmHg). The pupils on both sides were equally large (about 3 mm), the light reflex was dull in the left eye and disappeared in the right eye, the breathing was weak, the abdomen was swollen and soft, and the bowel sound was weak. Laboratory examination showed that white blood cell count was 4.81 × 10^9^/L (reference normal value is 4–10 × 10^9^/L), C-reactive protein (CRP) was 142.10 mg/L (reference normal value is <10 mg/L), potassium concentration was 2.56 mmol/L (reference normal value range is 3.6–5.2 mmol/L), procalcitonin (PCT) was 81.51 ng/ml (reference normal value range is <0.1 ng/ml), myoglobin (MB) was 2,906.44 ng/ml (reference normal range is 25–72 ng/ml), N-terminal brain natriuretic peptide was 1,030 pg/ml (reference normal range is <125 pg/ml), cardiac troponin I was 0.2667 ng/ml (reference normal range is 0–0.04 ng/ml), indicating a potentiality of bacterial infection and sepsis (Vijayan et al., 2017). Then, the antibiotic treatment was carried out, with imipenem/cilastatin sodium administered at a dose of 1,000/1,000 mg, by intravenous infusion. On December 30, 2021, the patient experienced a rapid drop in blood pressure and recovered after rescue at 2:45. To save the patient more effectively, deep venous catheterization, peripheral arterial catheterization, and temporary central venous catheterization for blood purification had been carried out during 5:50 and 5:51. At 10:34, fiberoptic bronchoscopy and bronchial aspiration were carried out. At 18:00, the laboratory examination showed an elevated PCT level (741.33 ng/ml vs. 81.51 ng/ml) and meropenem was given to the patient at a dose of 1.0 g every 8 h by intravenous infusion to control the infection. On December 31, 2021, a laboratory examination was conducted again to verify the patient’s signs. The patient was considered to be suffering from an uncontrolled bacterial infection based on the physical examination and a significant increase in white blood cell count (17.91 × 10^9^/L vs. 7.39 × 10^9^/L, reference normal range is 4–10 × 10^9^/L) and CRP (267.07 mg/L vs. 184.48 mg/L) (data of December 31 vs. data of December 30). Then, the ascitic fluid and blood of the patient were cultured in the clinical laboratory. A monoinfection of anaerobic bacteria was found based on the blood cultures. After many times of emergency treatment, the patient soon died of multiple organ failure and bacterial infection.

### Identification of the Isolate by Culture With MALDI–TOF MS

Ascitic fluid and blood cultures were carried out according to the principles and procedure guidelines for blood cultures raised by the Clinical and Laboratory Standards Institute (CLSI). Briefly, 10 ml of whole blood or ascitic fluid was separately injected into the BacT/ALERT culture media anaerobic bottle and then into the aerobic bottle (bioMérieux, Marcy I’É toile, France). Then, the bottles were put into the BacT/ALERT 3D Microbial Identification System (bioMérieux, Marcy I’É toile, France) for shaking culture at 35°C. The microorganism-positive bottle was then subcultured into the Columbia blood agar and cultured at 35°C for at least 48 h (put into the anaerobic bag or not). Pure colonies of the bacteria on the Columbia blood agar plate were selected for identification by a MALDI–TOF MS system, VITEK MS RUO IVD library (v3.2) (bioMÉrieux, Marcy I’É toile, France), following the manufacturer’s instruction.

### Antimicrobial Susceptibility Testing

E-test methods were used to determine the minimum inhibitory concentrations (MICs) of various antibiotics against the isolated organisms according to the 2021 CLSI criteria (Humphries et al., 2021). The following seven antibiotics were tested: penicillin G, vancomycin, ampicillin, ceftriaxone, meropenem, clindamycin, and chloramphenicol.

### Whole-Genome Sequencing of SAHP1

WGS is a valuable and most accurate tool for the definitive molecular identification of bacterial isolates (Tohya et al., 2022). The pure colonies on the plate were harvested and sent to Guangzhou IGE Biotechnology Ltd., where DNA sequencing was performed on the PromethION 48 platform developed by Oxford Nanopore Technologies using the protocol described elsewhere for WGS (Lu et al., 2016). After genome assembly, correction, and optimization, the final genome of the human blood isolate (designated as SAHP1) was obtained. Coding proteins of SAHP1 were annotated in Clusters of Orthologous Genes (COG), Kyoto Encyclopedia of Genes and Genomes (KEGG), Gene Ontology (GO), NCBI Reference Sequence (RefSeq), Pfam, and TIGR-defined protein families (TIGRFAMs) databases.

### Molecular Identification of SAHP1 Based on BLAST, Phylogenetic Analysis, Average Nucleotide Identity, Average Amino Acid Identity Calculation, and *In Silico* DNA-DNA Hybridization

For further molecular identification of SAHP1, we performed a set of analyses including the online BLAST (http://blast.ncbi.nlm.nih.gov/Blast.cgi), phylogenetic analysis based on the 16S ribosomal RNA (rRNA) gene, and multilocus sequence analysis (MLSA) to resolve its relationship with other bacterial species (Song et al., 2003; Glaeser and Kämpfer, 2015; Sayers et al., 2021). Average Nucleotide Identity (ANI), Average Amino Acid Identity (AAI) calculation, and *in silico* (also known as digital) DNA-DNA hybridization (iDDH or dDDH) were performed to research whether SAHP1 could be assigned as a new bacterial species (Richter and Rosselló-Móra, 2009; Auch et al., 2010; Thompson et al., 2013; Kim et al., 2014). Reference 16S rRNA gene sequences of the genus *Peptoniphilus* bacteria (all 30 species) were obtained from the NCBI database based on the accession numbers indicated on the website of List of Prokaryotic names with Standing in Nomenclature (LPSN) (https://lpsn.dsmz.de/) (Meier-Kolthoff et al., 2022). These sequences were used for a rough phylogenetic analysis because they were incomplete (~1,300 bp after trimming, 85% of the length of the full 16S rRNA gene for genus *Peptoniphilus*). The whole-genome sequences of the reference/type *Peptoniphilus* strains (19 strains belonging to 16 species) and a strain (*Anaerococcus degeneri* strain FDAARGOS1538) belonging to the family *Peptoniphilaceae* were obtained from the NCBI dataset for genomes (https://www.ncbi.nlm.nih.gov/datasets/genomes/). Full 16S rRNA gene (~1,525 bp) and 6 housekeeping gene sequences (*rpoB*, *gyrA*, *dnaA*, *recA*, *rplE*, and *groL*) of these strains were obtained based on the genome annotation of the NCBI dataset. Genome sequences and gene locus information of the analyzed strains are shown in **Supplementary Table S1**. An unrooted phylogenetic tree of the 16S rRNA gene and the 6 housekeeping genes of SAHP1 and the reference bacterial strains was constructed using MEGA X, inferring the evolutionary history using the neighbor-jointing (NJ) method (Kumar et al., 2018). Bootstrap values were estimated using 1,000 replications. ANI between the two paired strains was obtained by ANI calculator using the OrthoANIu algorithm, an improved iteration of the original OrthoANI algorithm, which uses USEARCH instead of BLAST (Yoon et al., 2017). AAI between the two paired strains was obtained by the CompareM packages (https://github.com/dparks1134/CompareM), which used DIAMOND to perform sequence similarity searches, and gene calling is performed using Prodigal (Hyatt et al., 2010; Yoon et al., 2017). The iDDH was performed using Genome-to-Genome Distance Calculator (GGDC), which could yield the highest correlations with traditional DDH, and values generated by the Genome BLAST Distance Phylogen (GBDP) formula *d_4_* are recommended as the standard (Meier-Kolthoff et al., 2013; Meier-Kolthoff et al., 2022).

### SAHP1 Phylogenetic Inference, Gene Family Construction, and Collinearity Analysis With Its Closest Relative Bacterial Species

To uncover the phylogenetic relationship between SAHP1 and other relative bacterial species, the genome sequences of SAHP1 and 19 other reference *Peptoniphilus* strains we mentioned above were uploaded to the Type (Strain) Genome Server (TYGS) inference for a whole genome-based taxonomic analysis (Meier-Kolthoff et al., 2022). The genomes were compared against all type strain genomes available in the TYGS database *via* the MASH algorithm (Ondov et al., 2016), and close relative type strains were obtained from the TYGS database and auto-implemented in further analysis. Pairwise comparisons of the genome sequences were conducted using the GBDP approach, and intergenomic distances were inferred under the algorithm “trimming” (Meier-Kolthoff et al., 2013). The resulting intergenomic distances were used to infer a balanced minimum evolution tree with branch support *via* FASTME 2.1.6.1 including Subtree Pruning and Regrafting (SPR) postprocessing (Lefort et al., 2015). Branch support was inferred from 100 pseudo-bootstrap replicates each.

For comparative analyses of the orthologous and exclusive genes between SAHP1 and the closest relative genomes, OrthoVenn2, an OrthoMCL-based method that uses DIAMOND (v0.9.24) instead of BLASTP or UBLAST to perform the all-against-all protein sequence comparison, was used (Xu et al., 2019). Collinearity of the conserved and highly orthologous genomic regions was determined and plotted among SAHP1 and its close relative *Peptoniphilus* species, reference strain KhD-2 (*P. vaginalis*), and NCTC13077 (*P. harei*) by using Mauve software (version 2.3.1) with default parameters (Darling et al., 2004).

## Results

### Characteristics of SAHP1 in the Clinical Laboratory

**Figure 2:**
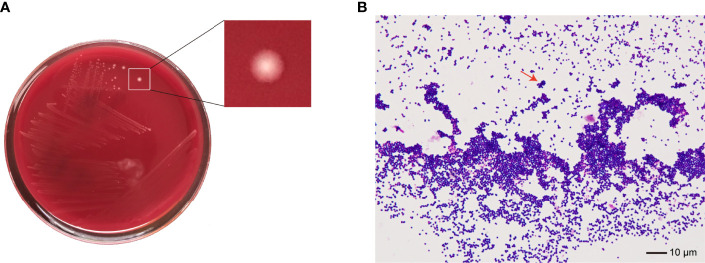
The phenotype of SAHP1. **(A)** Bacterial colonies on the Columbia blood agar after 48 h of incubation under anaerobic conditions. **(B)** Gram staining of bacteria from a positive aerobic blood culture showed Gram-positive cocci (×1,000 magnification) (arrowhead).

**Figure 3:**
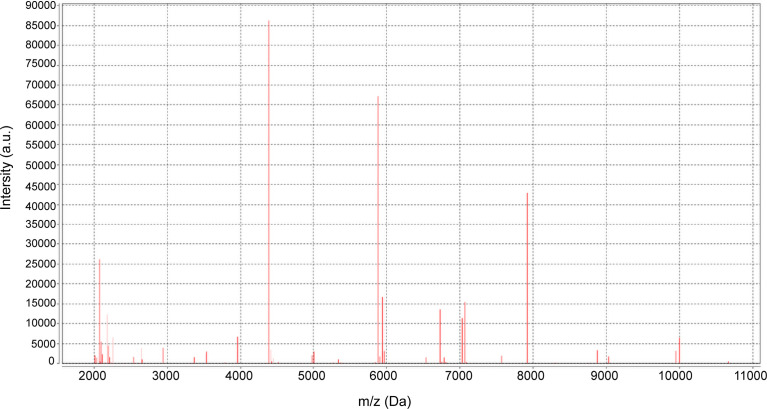
The spectrum of strain SAHP1 *via* the VITEK MS MALDI–TOF MS.

No bacteria were cultured from the patient’s ascitic fluid. The BacT/ALERT 3D Microbial Identification System reported positivity for microorganisms in the anaerobic bottle after 21 h and 37 min of culturing. Anaerobic culture of blood in Columbia agar plates in the anaerobic bag after 48 h revealed pinpoint, smooth, glistening white colonies with blur edges (**Figure 2A**). Gram staining showed Gram-positive cocci (**Figure 2B**). In contrast, the aerobic bottle remained negative after more than 48 h of culture incubation. VITEK MS analysis showed SAHP1 as *P. asaccharolyticus* based on a confidence level of 99.9%, and the spectrum of strain SAHP1 is shown in **Figure 3**. The SAHP1 was susceptible to all antimicrobials tested with variable MICs obtained: penicillin G (0.25 mg/L), vancomycin (0.125 mg/L), ampicillin (0.094 mg/L), ceftriaxone (0.125 mg/L), meropenem (0.004 mg/L), clindamycin (0.75 mg/L), and chloramphenicol (1 mg/L) (**Supplementary Figure S1**).

### Molecular Identification of SAHP1 Revealed It as a Novel *Peptoniphilus* Species That Has a Close Relationship with *P. vaginalis* and *P. harei*

**Figure 4:**
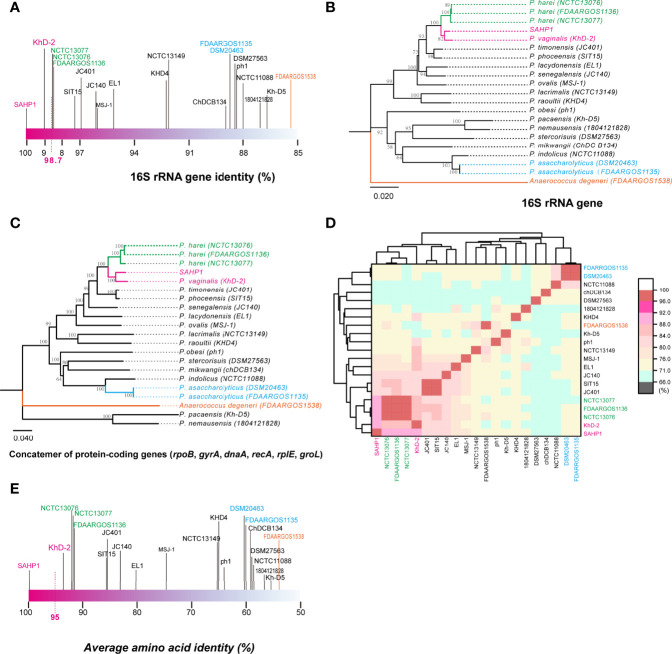
The 16S rRNA gene identity analysis and phylogenetic, ANI, and AAI analyses of SAHP1 with other reference strains in the genus *Peptoniphilus*. **(A)** The 16S rRNA gene identities between SAHP1 and other reference strains of the genus *Peptoniphilus.* Magenta indicates the highest identity of the 16S rRNA gene with SAHP1. Green indicates *P. harei* strains, while blue indicates *P. asaccharolyticus* strains, and orange indicates the *Anaerococcus degeneri* strain that belongs to the same family, the *Peptoniphilaceae*, as those *Peptoniphilus* strains. **(B)** Phylogenetic analysis of full 16S rRNA. **(C)** The MLSA-based phylogenetic tree of strain SAHP1 and other *Peptoniphilus* strains. The tree is based on six housekeeping protein-coding genes. Phylogenetic inference was performed with MEGA X. The bootstrap values of 1,000 replications display the significance of each branch, and those higher than 50% are shown in the taxa clustered in the tree. The tree is drawn to scale, with branch lengths measuring in the number of substitutions per site. **(D)** ANI between SAHP1 and different *Peptoniphilus* strains, and all showed a <92% ANI; the cluster diagram of ANI showed a close relationship among SAHP1 and *P. vaginalis* KhD-2 and the three *P. harei* strains. **(E)** AAI between SAHP1 and other reference strains. Green indicates *P. harei* strains, while blue indicates *P. asaccharolyticus* strains, and orange indicates the *Anaerococcus degeneri* strain that belongs to the same family, the *Peptoniphilaceae*, as those *Peptoniphilus* strains.

In molecular identification *via* 16S rRNA gene BLAST against the nucleotide collection database using the default algorithm parameters, we found that SAHP1 exhibited a 16S rRNA similarity of 95.77%–100% with genus *Peptoniphilus* bacteria (top 20 hints, **Supplementary Figure S2A**). Phylogenetic analysis of partial 16S rRNA gene of SAHP1 and other 30 *Peptoniphilus* species type strains revealed that SAHP1 was closest to *P. vaginalis* strain KhD-2 and *P. harei* strain DSM10020 that had ever been isolated from the human female genital tract or other human clinical specimens (Murdoch et al., 1997; Diop et al., 2016; Diop et al., 2019). The three strains clustered into the same group, and even the same subgroup (**Supplementary Figure S2B**). For accurate species identification, whole-genome sequences of 19 well-documented reference or type strains belonging to 16 species of *Peptoniphilus* were downloaded from NCBI datasets for genomes (https://www.ncbi.nlm.nih.gov/datasets/genomes/). These strains were taxonomy-checked based on the NCBI assembly database report. The full 16S rRNA gene sequence identities of SAHP1 and other strains are shown in **Figure 4A** and **Supplementary Table S2**. Briefly, SAHP1 showed a 99.02% similarity of 16S rRNA with *P. vaginalis* strain KhD-2, a 98.65%–98.69% similarity with three *P. harei* strains (NCTC13077, NCTC13076, FDAARGOS1136), and an 88.65% similarity with the two *P. asaccharolyticus* strains (FDAAROGS1135 and DSM20463). Given that 98.7% of 16S rRNA sequence identity is recommended to delineate a new species in the phylum *Firmicutes* without carrying out DNA-DNA hybridization (Stackebrandt, 2006; Diop et al., 2016), this result indicated that SAHP1 did not belong to *P. asaccharolyticus* and *P. harei* but might belong to *P. vaginalis*. A phylogenetic tree of 21 complete 16S rRNA sequences was constructed using the NJ method. Consistent with the 16S rRNA sequence comparison results, the SAHP1 clustered most closely with *P. vaginalis* strain KhD-2 (**Figure 4B**). Phylogenetic analyses based on the housekeeping gene sequences (single gene or trimmed 10,794-bp concatemers) also showed that SAHP1 formed a most closely cluster with *P. vaginalis* KhD-2 within the genus *Peptoniphilus* (**Figure 4C**, **Supplementary Figure S3**). To further species identification, we adopt more principles to identify SAHP1 as a strain belonging to an existing *Peptoniphilus* species. First, the ANI should be 95%–96% within SAHP1 and an existing species; second, the AAI should be >95% within SAHP1 and an existing species; ultimately, iDDH should show >70% similarity within SAHP1 and an existing species (Auch et al., 2010; Thompson et al., 2013; Chun et al., 2018). To our surprise, the highest ANI of SAHP1 against *Peptoniphilus* strains was only 91.93% (against *P. vaginalis* KhD-2), less than the same species threshold of 95%–96%, which indicates that SAHP1 did not belong to *P. vaginalis*, although the ANI heatmap showed a close relationship of SAHP1 with *P. vaginalis* (**Figure 4D**, **Supplementary Table S3**). Similarly, the highest AAI of SAHP1 against *Peptoniphilus* strains was only 93.51% (against *P. vaginalis* KhD-2), less than the threshold of 95% for the same bacterial species (**Figure 4E**, **Supplementary Table S4**). The iDDH of SAHP1 with the reference strains and known type strains in the TYGS database all showed a <70% identity, with the *P. vaginalis* KhD-2 having the highest identity (45.1%, 95% CI 42.5%–47.7%) (**Table 1**). Together, these results indicated that SAHP1 is a novel *Peptoniphilus* sp. that has the closest relationship with *P. vaginalis.* Here, we designated it as *Peptoniphilus septimus* sp. nov.

**Table 1:** iDDH of SAHP1 with other reference strains.

Query strain	Subject strain	iDDH (d0, in %)	95% CI (d0, in %)	iDDH (d4, in %)*	95% CI (d4, in %)	iDDH (d6, in %)	95% CI (d6, in %)	G+C content difference (in %)	Taxonomy check (by NCBI)
*SAHP1*	*P. vaginalis (KhD-2)*	66.3	[62.5–70.0]	45.1	[42.5–47.7]	62.7	[59.4–65.9]	0.35	OK
*SAHP1*	*P. harei (NCTC13076)*	59.6	[55.9–63.1]	35.4	[33.0–37.9]	53.6	[50.5–56.7]	0.83	OK
*SAHP1*	*P. harei (NCTC13077)*	66.3	[62.4–69.9]	35.0	[32.5–37.5]	58.4	[55.1–61.5]	0.16	OK
*SAHP1*	*P. harei (FDAARGOS1136)*	61.4	[57.7–65.0]	35.3	[32.9–37.8]	54.9	[51.8–58.0]	0.72	OK
*SAHP1*	*P. phoceensis (SIT15)*	52.6	[49.1–56.1]	26.8	[24.4–29.3]	44.5	[41.5–47.5]	3.38	OK
*SAHP1*	*P. timonensis (JC401)*	46.2	[42.9–49.7]	26.2	[23.9–28.7]	39.9	[36.9–42.9]	3.9	OK
*SAHP1*	*P. senegalensis (JC140)*	37.8	[34.5–41.3]	24.1	[21.8–26.6]	33.2	[30.3–36.3]	2.34	OK
*SAHP1*	*P. lacydonensis (EL1)*	27.3	[24.0–31.0]	23.2	[20.9–25.6]	25.3	[22.5–28.5]	4.68	OK
*SAHP1*	*P. ovalis (MSJ-1)*	19.2	[16.1–22.8]	20.5	[18.2–22.9]	18.6	[16.0–21.6]	3.97	OK
*SAHP1*	*P. raoultii (KHD4)*	14.7	[11.9–18.1]	22.3	[20.0–24.8]	14.9	[12.5–17.8]	2.70	OK
*SAHP1*	*P. lacrimalis (NCTC13149)*	14.7	[11.8–18.1]	32.9	[30.5–35.4]	15.1	[12.6–17.9]	4.24	OK
*SAHP1*	*P. obesi (ph1)*	14.6	[11.8–18.1]	35.4	[32.9–37.9]	15.1	[12.6–18.0]	4.44	OK
*SAHP1*	*P. asaccharolyticus (DSM20463)*	13.7	[11.0–17.1]	29.3	[26.9–31.8]	14.1	[11.7–16.9]	2.29	OK
*SAHP1*	*P. asaccharolyticus (FDAARGOS1135)*	13.8	[11.0–17.1]	29.5	[27.1–32.0]	14.2	[11.7–17.0]	2.18	OK
*SAHP1*	*P. nemausensis (1804121828)*	13.6	[10.9–17.0]	47.4	[44.8–50.0]	14.1	[11.7–17.0]	11.18	OK
*SAHP1*	*P. indolicus (NCTC11088)*	13.3	[10.5–16.6]	23.1	[20.8–25.6]	13.6	[11.2–16.4]	2.92	OK
*SAHP1*	*P. pacaensis (Kh-D5)*	13.1	[10.3–16.3]	39.6	[37.1–42.1]	13.5	[11.1–16.3]	14.81	OK
*SAHP1*	*P. mikwangii (ChDCB134)*	13.2	[10.5–16.5]	21.7	[19.5–24.1]	13.6	[11.2–16.3]	3.59	OK
*SAHP1*	*P. stercorisuis (DSM27563)*	13.2	[10.5–16.5]	18.1	[15.9–20.4]	13.5	[11.1–16.3]	6.75	OK
*SAHP1*	*Anaerococcus degeneri (FDAARGOS1538)*	13.1	[10.6–16.6]	34.1	[31.6–36.6]	13.7	[11.4–16.5]	0.66	OK
*SAHP1*	*P. grossensis (ph5)*	45	[41.6–48.4]	25.1	[22.7–27.5]	38.5	[35.5–41.5]	0.70	OK
*SAHP1*	*P. gorbachii (DSM 21461)*	44.8	[41.5–48.3]	24.8	[22.5–27.3]	38.3	[35.3–41.3]	3.19	OK
*SAHP1*	*P. urinimassiliensis (Marseille-P3195)*	13.2	[10.5–16.5]	35.8	[33.4–38.4]	13.6	[11.2–16.4]	15.11	OK
*SAHP1*	*P. rhinitidis (1-13T)*	26.2	[22.8–29.8]	23.0	[20.7–25.4]	24.4	[21.6–27.5]	4.72	Inconclusive
*SAHP1*	*P. olsenii (DSM 21460)*	16	[13.1–19.5]	22.2	[19.9–24.7]	16.1	[13.5–19.0]	4.59	N/A
*SAHP1*	*P. duerdenii (ATCC BAA-1640)*	14.6	[11.8–18.0]	32.8	[30.4–35.3]	15.0	[12.5–17.9]	0.37	Inconclusive
*SAHP1*	*P. koenoeneniae (DSM22616)*	14.2	[11.4–17.6]	30.9	[28.5–33.4]	14.6	[12.1–17.4]	3.26	N/A
*SAHP1*	*P. coxii (CCUG59622)*	13.8	[11.0–17.1]	37.8	[35.4–40.3]	14.2	[11.8–17.0]	9.99	Inconclusive
*SAHP1*	*P. ivorii (NCTC13079)*	12.7	[10.1–16.0]	30.4	[28.0–32.9]	13.2	[10.8–15.9]	17.52	Inconclusive
*SAHP1*	*P. tyrrelliae (CCUG59621)*	37.2	[33.8–40.7]	23.8	[21.5–26.3]	32.6	[29.7–35.7]	2.62	Inconclusive
*SAHP1*	*P. porci (35-6-1)*	28.0	[24.7–31.7]	23.6	[21.3–26.1]	26.0	[23.1–29.1]	3.37	Inconclusive

*Formula d_4_ (also known as GGDC formula 2) sums all identities found in high-scoring segment pairs (HSPs) divided by the overall. The results obtained from d_4_ are more robust and recommended as standard ones. N/A indicates not available. Cells filled with gray indicate that the genomic data of these strains were auto-implemented and from the TYGS database.

### Genomic Features of SAHP1 and Comparative Genomic Analysis

**Figure 5:**
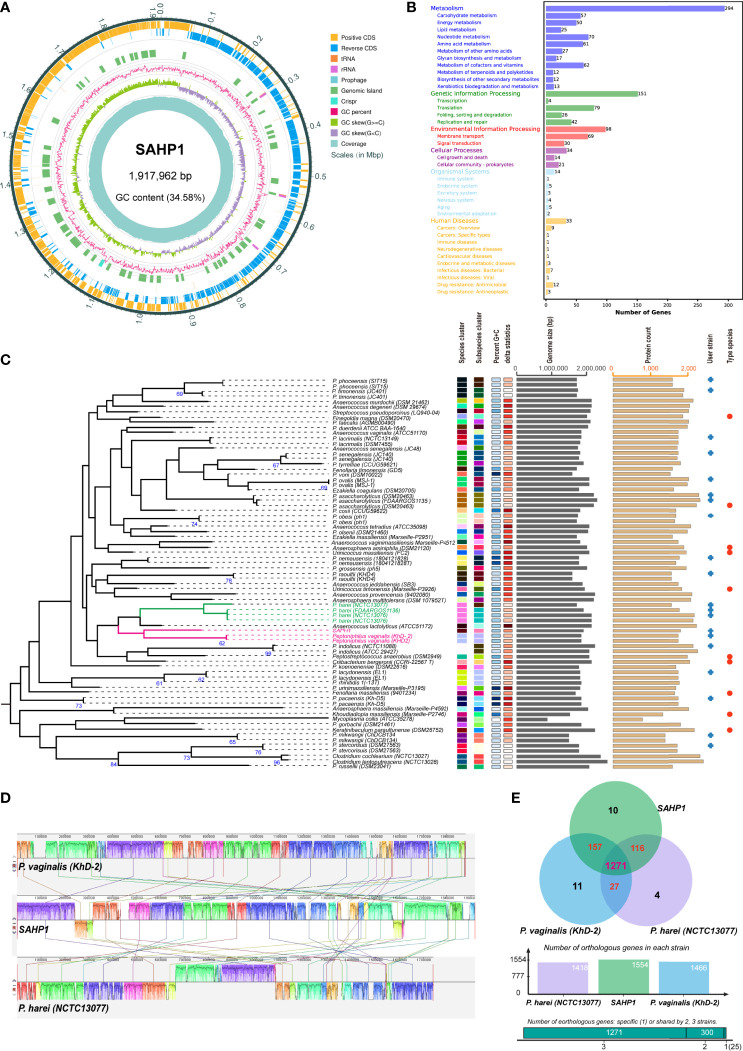
Genomic features of SAHP1 and comparative genomic analysis. **(A)** Graphical circular map of *P. septimus* strain SAHP1 genome. **(B)** Functional categorization of SAHP1 protein-coding genes based on the KEGG. **(C)** The GBDP tree of SAHP1 and other related strains using the genome data from the NCBI database (user strain) and those automatically implemented by the TYGS database. The tree was inferred with FastME 2.1.6.1 from GBDP distances calculated from genome sequences. The branch lengths are scaled in terms of GBDP distance formula d_5_. The numbers above the branches are GBDP pseudo-bootstrap support values >60% from 100 replications, with an average branch support of 29.8%. **(D)** Genome-to-genome alignment of *P. septimus* strain SAHP1, *P. vaginalis* strain KhD-2, and *P. harei* strain NCTC13077 using a progressive mauve software and *P. septimus* strain SAHP1 as the reference genome. Boxes with the same color indicate the syntenic regions. Boxes below the horizontal line indicate inverted regions. The colored locally collinear blocks (LCBs) show the conserved and highly similar genomic regions. The white areas inside the colored regions indicate sequence elements specific to one genome that are not aligned. The height of the similarity profile is present inside each block. The colored lines that connect LCBs represent translocations of homologous regions. Blocks above or below the horizontal bar indicate regions that aligned in the forward or reverse orientation, respectively. Rearrangements are shown by colored lines. **(E)** Venn diagram showing the number of orthologous protein-coding genes shared and unique between the three strains SAHP1, KhD-2, and NCTC13077.

The genome of *P. septimus* strain SAHP1 is composed of a circular chromosome of 1,917,962 bp (1 chromosome, with a clustered regularly interspaced short palindromic repeats (CRISPR) sequence, a prophage region, and without plasmid) with an overall 34.58% guanine-cytosine (GC) content and 1,804 protein-coding genes, 9 rRNA genes, 43 transfer RNA (tRNA) genes, 27 regulatory noncoding RNA (ncRNA) genes, and 9 other ncRNA genes (**Figure 5A**, **Table 2**). The number of protein-coding genes annotated by the COG, KEGG, GO, Refseq, Pfam, and TIGRFAMs database for SAHP1 is shown in **Supplementary Table S5**. In this study, 1,745 protein-coding genes (95.07%) have predicted functions by COG, while 59 have unknown functions. The properties and the annotated features by COG and GO are summarized in **Supplementary Figure S4**. None of the virulence factors in the SAHP1 was found in the Virulence Factor Database (VFDB). The KEGG only showed that 33 SAHP1 genes were associated with human disease, of which only 7 might take part in the bacterial infection (**Figure 5B**), indicating that *P. septimus* SAHP1 might be a normal microbiota for human beings. Five antibiotic-resistant genes of *P. septimus* SAHP1 were found in the Comprehensive Antibiotic Research Database (CARD), including tetM, ErmA, aad (Puapermpoonsiri et al., 1996), SAT-4, and APH (3’)-IIIa, indicating potential tetracycline, macrolide-lincosamide-streptogramin B, aminoglycoside, and streptothricin resistance in SAHP1 (**Supplementary Table 6**).

**Table 2:** General features of the genome of *P. septimus* strain SAHP1.

Parameters	Values (n)	% of total*
Genome size (bp)	1,917,962	100
DNA coding region (bp)	1,701,678	88.72
G+C content (bp)	663,137	34.58
Total genes	1,892	100
ncRNA	88	4.65
rRNA	9	10.23
tRNA	43	48.86
Regulatory ncRNA	27	30.68
Other ncRNA	8	9.09
Protein-coding genes	1,804	95.35
Genes with function prediction	1,745	95.07
Genes assigned to COG	1,394	77.27
Genes assigned to KEGG	960	53.21
Genes associated with human disease	33	1.83
Genes associated with bacterial infection	7	0.39
Genes associated with metabolism	294	16.30

*The total is based on either the size of the genome (base pairs) or the total number of genes or protein-coding genes in the annotated genome.

The GBDP tree based on the whole-genome sequences of the SAHP1 and those closely related type strains in the TYGS database is shown in **Figure 5C**. The *P. septimus* SAHP1 still showed a close genetic relationship with *P. vaginalis* and *P. harei*—the closest with *P. vaginalis.* The *P. septimus* SAHP1 genome is larger than that of the *P. vaginalis* KhD-2 (1,877,211 bp) and *P. harei* NCTC13077 (1,739,102 bp). The collinearity analysis is consistent with the close relatedness among the above three strains (**Figure 5D**). OrthoMCL analysis of the orthologous genes among the three strains showed that 1,271 genes make up the core genome, occupying 79.64% (1,271/1,596) of the pan-genome and 78.96%–89.63% of each genome (**Figure 5E**). In this study, 91.89% (1,428 genes) and 89.25% (1,387 genes) of *P. septimus* SAHP1 genes have orthologs in the *P. vaginalis* KhD-2 and *P. harei* NCTC13077, respectively, and only 10 genes (0.82%) are unique to the *P. septimus* SAHP1 genome (**Figure 5E**).

## Discussion

*Peptoniphilus* may always act as normal microbiota that inhabit human skin and mucosal surfaces. However, many local infections caused by *Peptoniphilus* were found, indicating that they may also act as common pathogens to human beings in some circumstances. The uncommon cause of bacteremia in humans by *Peptoniphilus* confirmed their potential to cause poor clinical outcomes and high rates of mortality, as it was shown that 20% of fatal outcomes were caused by bloodstream infections (Brown et al., 2014). The case we reported in the present study had several unique and interesting clinical characteristics. First, *Peptoniphilus* always acts as one of the causative agents in polymicrobial infections in humans, such as ulcers and osteoarticular and soft tissue infections, while our case revealed a monoinfection of the bloodstream caused by *Peptoniphilus* bacteria, which was rarely reported previously (Brown et al., 2014; Wan et al., 2021). Second, the patient was suffering from a genital tract cancer, cervical cancer, and the causative agent of bloodstream infection was a genetically *P. vaginalis*-like bacterium, which may inhabit the human female genital tract as *P. vaginalis* does, acts as a commensal of the human vagina, and is frequently associated with bacterial vaginosis (Verma et al., 2017). The bacterial characteristics and the case made us deem a possible infection pathway from the present case. The invasive operations in the genital tract of the patient during examinations and treatment (e.g., removal of the IUD, colposcopy, and ureteral dilatation) or the tumor tissue damage and bleeding that were very common in patients with advanced cervical cancer might facilitate the bacteria inhabiting the genital tract and entering the bloodstream (Eleje et al., 2019). The immunosuppressive state caused by the tumor and chemotherapy treatment may impair the neutrophils, monocytes, macrophages, and host immune response to the bacteria in the tissue and also facilitate the bacteria entering the bloodstream (Minasyan, 2017). After entering the bloodstream, bacteria move with blood flow. By mechanisms against reactive oxygen species that are released from the main bactericidal cells in the blood, erythrocytes, the bacteria survive oxidation on the surface of erythrocytes and proliferate and in turn trigger a systemic inflammatory response of the host to the infection, which leads to multifaceted disruption of the finely tuned immunological balance of inflammation and anti-inflammation and finally causes septic shock-like symptoms in the patient who has been seen at the end of the case of the present study (Jarczak et al., 2021). However, this could not exclude other infection pathways, such as the bacteria inhabiting the skin or gastrointestinal tract and migrating to the blood through other ways (e.g., deep venous catheterization, peripheral arterial catheterization, and temporary central venous catheterization). Third, this was the first case of bloodstream monoinfection by *Peptoniphilus* bacteria found in a cervical cancer patient and contributed to the patient’s death, and the novel *Peptoniphilus* species we designated in the present study was genetically close to *P. vaginalis.* The antimicrobial susceptibility of the bloodstream infection agent *P. septimus* SAHP1 showed that it was susceptible to the tested broad-spectrum antibiotics, including penicillin G, vancomycin, ampicillin, ceftriaxone, meropenem, clindamycin, and chloramphenicol. However, genome research revealed the probable resistance of SAHP1 to tetracycline, macrolide-lincosamide-streptogramin B, aminoglycoside, and streptothricin. Thus, clinicians need to pay attention to the possible antibiotic resistance of this bacterium. The E-test showed that SAHP1 was sensitive to clindamycin (MIC = 0.75 mg/L), which seems to be different with the genome research result based on the presence of macrolide-lincosamide-streptogramin B resistance gene, named *ermA*. The ErmA protein dimethylates the A2058 residue of 23S rRNA and in turn impairs the binding of macrolides, lincosamides, and streptogramin B, which accounts for the cross-resistance to these drugs (Petinaki and Papagiannitsis, 2018). Thus, the first is probably that ErmA in the SAHP1 has no or very weak methylase activity due to a relatively low nucleotide sequence identity (82.5% with *Staphylococcus aureus* strain N315 *ermA* gene) and amino acid sequence identity (81.0% with *Staphylococcus aureus* strain N315 ErmA protein). Second, the *ermA* gene expression in the SAHP1 may be inducible, which makes SAHP1 susceptible to 16-membered ring macrolides, lincosamides, and streptogramin B in the absence of 14- and 15-ring macrolides (Petinaki and Papagiannitsis, 2018). Although SAHP1 was shown to be susceptible to meropenem *in vitro*, we found that meropenem might not control the SAHP1 infection in the patient based on the case record. Likely reasons included insufficient meropenem treatment time or the SAHP1 might present in the biofilm or present as L-form in the bloodstream that was common in the sepsis patient, which made it resistant to most of the antibiotics *in vivo* (Minasyan, 2019).

Correctly identifying pathogens is of crucial importance in clinical microbiology and epidemiology research. *Peptoniphilus* spp. are often misidentified by using biochemical methods (Veloo et al., 2011). Due to the limited quantity and accuracy of the MALDI–TOF MS database, the mass spectrometry results may be uncertain when encountering *Peptoniphilus* spp. Evaluations of MALDI–TOF MS for the identification of anaerobic bacteria showed 84%–94.8% accuracy (Barba et al., 2014; Garner et al., 2014; Rodríguez-Sánchez et al., 2016; Li et al., 2019), indicating a dilemma in identifying *Peptoniphilus* species by using the MALDI–TOF MS (Barberis et al., 2022). A previous study by Wan et al. (2021) found misidentification of *P. harei* strain to *P. asaccharolyticus* by the VITEK MS system. We also found a species-level misidentification of SAHP1 caused by the absence of *P. septimus* in the database, emphasizing the importance and urgency of implementing the standard mass spectrum of *P. septimus* in the database.

With the presence of the 16S rRNA gene in almost all bacteria, the role of the “molecular time scale” and the convenience of analysis make it the most popular target for bacterial phylogeny and taxonomy (Janda and Abbott, 2007). Despite the full 16S rRNA gene providing better taxonomic resolution (Johnson et al., 2019), its resolution at the bacterial species level is not satisfying; even if the bacterial strains have >99.7% 16S rRNA gene identity, they may also belong to different species in some cases (e.g., some *Shigella* vs. *Escherichia* strains) (Fox et al., 1992; Devanga Ragupathi et al., 2018). Thus, many other indices that utilized genome data to resolve the genetic relatedness were used for bacterial species identification and taxonomy, including the ANI, AAI, and iDDH (Richter and Rosselló-Móra, 2009; Auch et al., 2010; Thompson et al., 2013; Kim et al., 2014). In the present study, the full 16S rRNA gene of SAHP1 has 99.02% identity with the type strain of *P. vaginalis*. However, taxonomy based on the genomic data (ANI = 91.93%, AAI = 93.51%, and iDDH = 45.1% with its closest relative species type strain KhD-2; all values have not reached the threshold that could be identified as the same species) revealed that SAHP1 was a novel *Peptoniphilus* species. Given that the BLAST results showed that the SAHP1 16S rRNA gene has higher identities (all >99%) with *P. harei* strain TID-12, DCW_SL_32C, and *P. asaccharolyticus* strain 1212-10216, we suspected that the misidentification of *Peptoniphilus* strains may be very common, underlining the importance of molecular species identification methods (e.g., WGS) other than the 16S rRNA gene sequencing.

Genomic information revealed that *P. septimus* strain SAHP1 had none of the virulence factors that were found in the VFDB, and the small number of genes implicated in the infection pathway based on the KEGG annotation implies it as a commensal and opportunistic pathogen, like many other GPACs (Chen et al., 2005; Murphy and Frick, 2013). This could also be deduced from the present case: a woman with a malignant genital tract tumor, an immunosuppressive state, etc. The SAHP1 had a comparable genome size, number of protein-coding genes, and GC contents (0.16%–0.83% difference, **Table 1**) with its close relatives (*P. vaginalis* and *P. harei*). Similar genetic and microbiological properties may have been shared among these species, which were implicated in the collinearity and OrthoMCL analysis, which also implied that the bacteria of the genus *Peptoniphilus* might be relatively evolutionarily conservative (Li et al., 2003). However, a mosaic structure of the GBDP tree (mainly consisting of genus *Peptoniphilus* species) could be observed, which was filled with some other bacterial species in the family *Peptoniphilaceae* such as *Anaerococcus lactolyticus*, *Finegoldia magna*, *Urinicoccus massiliensis*, and *Khoudiadiopia massiliensis*. This may indicate a history of non-vertical inheritance of genetic material in these species and the probable requirement of changes in the nomenclature in the species of the family *Peptoniphilaceae* (Katz, 2021).

## Conclusions

In summary, we have reported a case of a cervical cancer patient receiving chemotherapy monoinfected with *P. vaginalis*-like bacteria in the bloodstream. Based on the probable similar microbiological characteristics of *P. septimus* to other GPACs, clinicians need to pay attention to those patients with genital tract tumors and must include anaerobic blood cultures as part of their blood culture procedures to avoid infection and carry out early antibiotic therapy. The identification procedures of the novel species *P. septimus* in the present study reveal the limited and insufficient resolution of MALDI–TOF MS in identifying *Peptoniphilus* bacteria in the clinical laboratory. Missed identification and misidentification of *Peptoniphilus* species might occur many times in the clinical laboratory. The 16S rRNA gene sequencing is required in the clinical laboratory but not adequate, and whole genome-based identification of *Peptoniphilus* bacteria is recommended, as it could provide more detailed information (e.g., AAI, ANI, iDDH) when analyzed with other strains and could catch some “hidden” novel species. These molecular methods may help to better estimate real GPAC prevalence and pathogenicity in the clinic. Genomic features of SAHP1 and comparative genomic analysis among *P. septimus*, *P. vaginalis*, and *P. harei* further confirmed the close genetic relationship of the three bacterial species and might imply comparable microbiological characteristics and pathogenies among them.

## Data Availability Statement

The datasets presented in this study can be found in online repositories. The names of the repository/repositories and accession number(s) can be found below: https://www.ncbi.nlm.nih.gov/nuccore/CP097885.

## Author Contributions

X-YZ designed the study and wrote the manuscript. HW, J-LY, CC, YZ, MC, JQ, and ST collected the data of the case, HW and YZ performed the antibiotic subspeciality research, and J-LY cultured the bacteria for WGS and collected the genomic sequence of other bacterial strains. X-YZ analyzed the data and performed graphing and data interpreting. All authors contributed to the article and approved the submitted version.

## Funding

This work was supported by the National Natural Science Foundation of China (Grant No. 31870001), and Shenzhen Science and Technology Innovation Commission Fund (Project No. JCYJ20210324122802006) to X-YZ.

## Conflict of Interest

The authors declare that the research was conducted in the absence of any commercial or financial relationships that could be construed as a potential conflict of interest.

## Publisher’s Note

All claims expressed in this article are solely those of the authors and do not necessarily represent those of their affiliated organizations, or those of the publisher, the editors and the reviewers. Any product that may be evaluated in this article, or claim that may be made by its manufacturer, is not guaranteed or endorsed by the publisher.
