# Supplementary material for: Identification of Peptoniphilus vaginalis-Like Bacteria, Peptoniphilus septimus sp. nov., From Blood Cultures in a Cervical Cancer Patient Receiving Chemotherapy: Case and Implications
Source: Front Cell Infect Microbiol. 2022 Jul 8;12:954355. doi: 10.3389/fcimb.2022.954355 (PMC9307962; doi:10.3389/fcimb.2022.954355)
Supplement: Supplementary file 2 [file DataSheet_2.pdf]

A

Description	Taxid	Max Score	Total Score	Query Cover	E value	Acc. Len	Accession	Per. ident
P. harei partial(TID-12)	54005	2724	2724	96%	0	1475	LN871839.1	100
P. harei (1-DE-D40-21)	54005	2451	2451	87%	0	1329	MT903189.1	100
P. harei (DCW_SL_32C)	54005	2567	2567	91%	0	1394	MK424035.1	99.93
P. asaccharolyticus (unnamed strain)	1258	2553	2553	91%	0	1394	DQ986463.1	99.64
P. asaccharolyticus (1312-10216)	1258	2567	2567	92%	0	1414	KP944180.1	99.44
P. asaccharolyticus (W16018C2)	1258	2573	2573	93%	0	1424	KP944181.1	99.37
P. harei (FDAARGOS1012)	54005	2700	8091	100%	0	1791970	CP066287.1	98.62
P. harei (FDAARGOS1136)	54005	2700	8091	100%	0	1929566	CP068103.1	98.62
P. harei (NCTC13077)	54005	2700	8091	100%	0	1739102	LR134524.1	98.62
P. harei (DCW_SL_25A)	54005	2549	2549	94%	0	1440	MK424030.1	98.61
P. harei (DSM10020)	54005	2604	2604	96%	0	1476	NR_026358.1	98.51
P. gorbachii (WAL10418)	411567	2398	2398	92%	0	1404	NR_115885.1	97.51
P. phoceensis (SIT15)	1720298	2499	2499	97%	0	1488	NR_144732.1	96.98
P. grossensis (ph5)	1118057	2481	2481	97%	0	1482	NR_125605.1	96.9
P. indolicus (GIFU7848)	33030	2289	2289	90%	0	1372	NR_115509.1	96.65
P. faecalis (AGMB00490)	2731255	2473	2473	98%	0	1498	MT396160.1	96.46
P. ovalis (MSJ-1)	2841503	2218	2218	88%	0	1354	MZ310594.1	96.43
P. senegalensis (JC140)	1033744	2272	2272	90%	0	1386	NR_125592.1	96.26
P. tyrrelliae (RMA19911)	755171	2386	2386	95%	0	1463	NR_117555.1	96.11
P. lacydonensis (EL1)	1673725	2355	2355	95%	0	1459	NR_157682.1	95.77

B

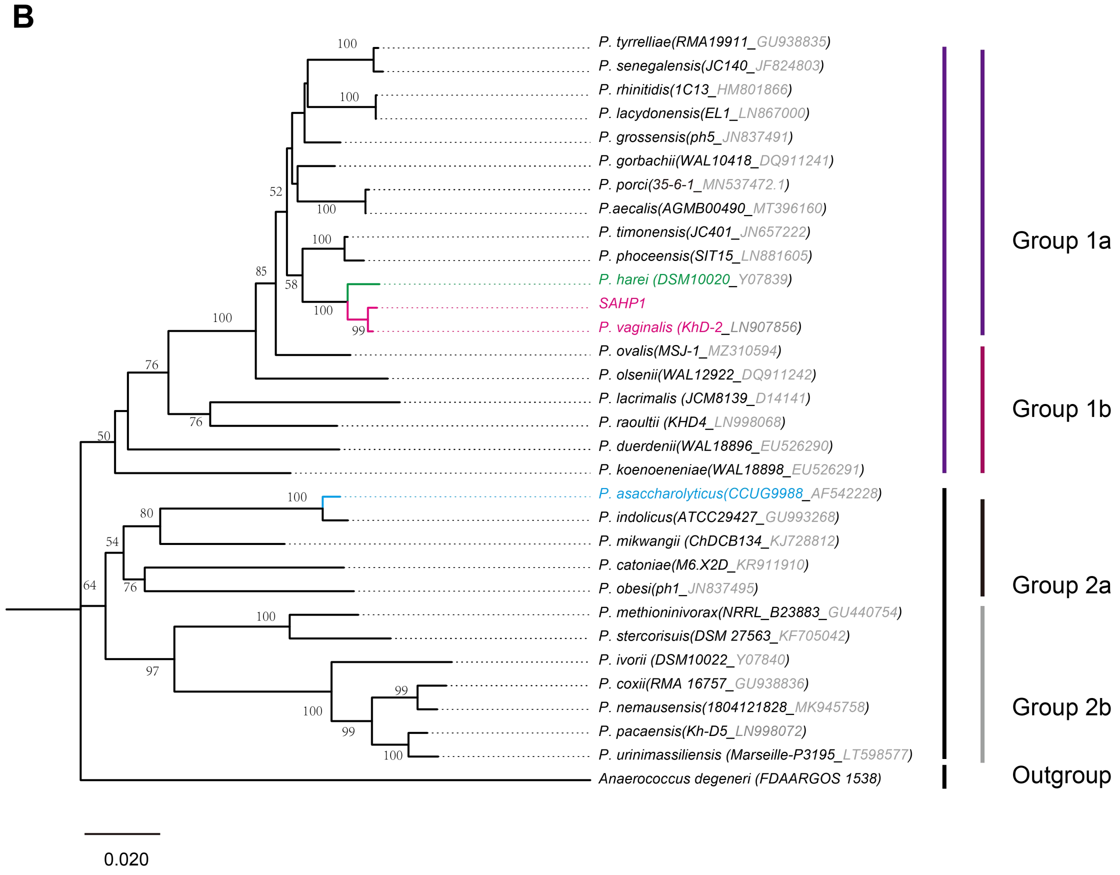

Supplementary Figure 2. Alignment of 16S rRNA gene and phylogenetic analysis of 16S rRNA gene with *Peptoniphilus* species listed in the LPSN. A. Alignment of 16S rRNA gene was conducted using nucleotide BLAST program by default parameters, against the nucleotide collection database. The top 20 identities are shown, and uncultured or species undefined strains were removed. **B.** Phylogenetic analysis of SAHP1 16S rRNA gene with those of type strains belonging to different *Peptoniphilus* species listed in the LPSN. GenBank accession numbers of the 16S rRNA (most of which were partial 16S rRNA genes) of the strains are colored gray. 16S rRNA gene sequence of *Anaerococcus degeneri* strain FDAARGOS1538 was obtained by searching locus ID: LDJ82_RS02495. Phylogenetic groups and subgroups are shown. The related *Peptoniphilus* species are colored. Phylogram was constructed using the neighbor-joining method with 1000 bootstrap replicates. Bootstrap values higher than 50% are shown.
