# Supplementary material for: Identification of Peptoniphilus vaginalis-Like Bacteria, Peptoniphilus septimus sp. nov., From Blood Cultures in a Cervical Cancer Patient Receiving Chemotherapy: Case and Implications
Source: Front Cell Infect Microbiol. 2022 Jul 8;12:954355. doi: 10.3389/fcimb.2022.954355 (PMC9307962; doi:10.3389/fcimb.2022.954355)
Supplement: Supplementary file 3 [file DataSheet_3.pdf]

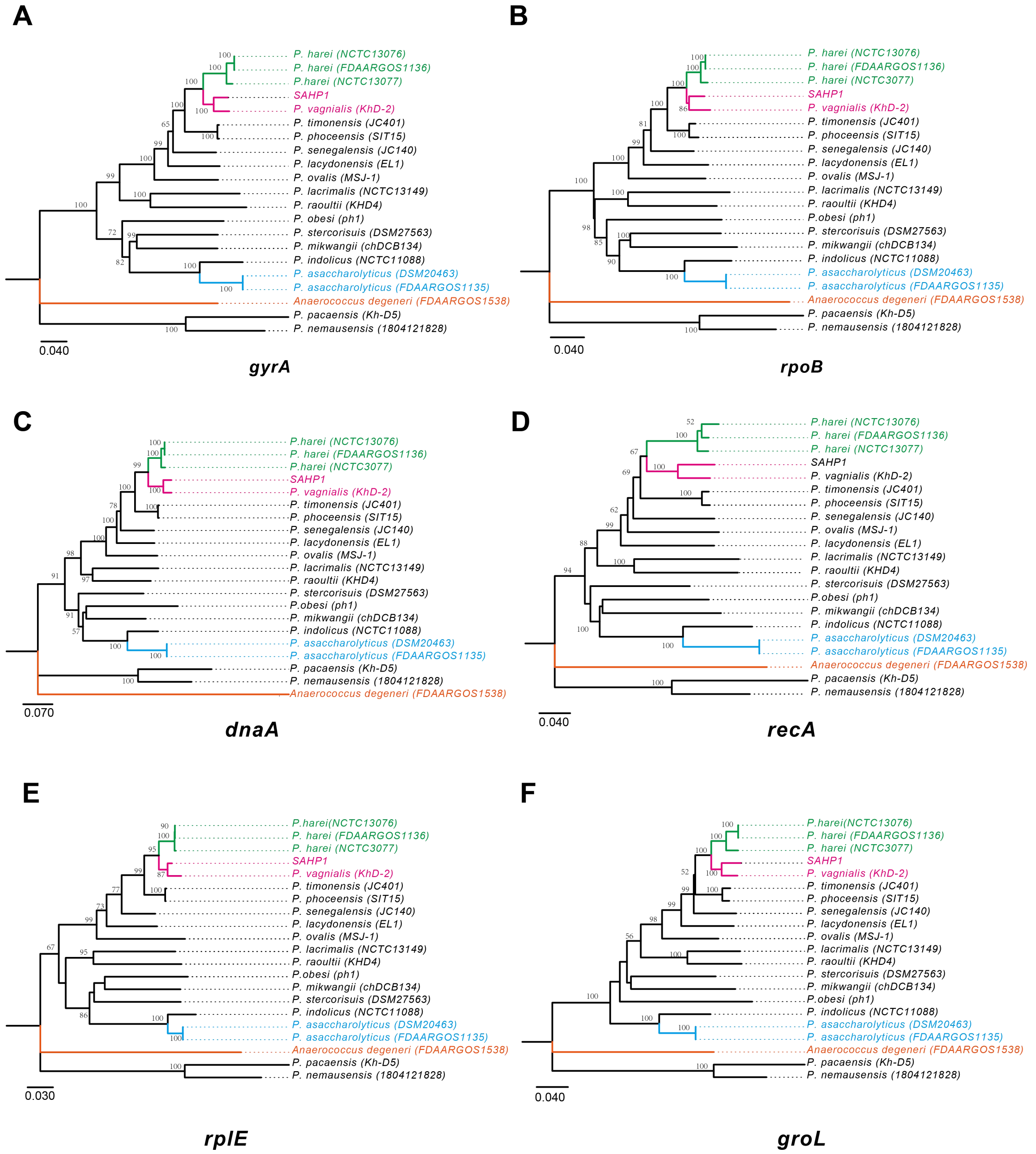

Supplementary Figure 3. Phylogenetic tree of strain SAHP1 with other reference strains belonging to *Peptoniphilus* species. The tree is based on 6 housekeeping protein-coding genes independently (A-F). Neighbor-joining phylogenetic inference was performed. The bootstrap values of 1,000 replications display the significance of each branch, and those higher than 50% are shown.
