# Supplementary material for: Identification of Peptoniphilus vaginalis-Like Bacteria, Peptoniphilus septimus sp. nov., From Blood Cultures in a Cervical Cancer Patient Receiving Chemotherapy: Case and Implications
Source: Front Cell Infect Microbiol. 2022 Jul 8;12:954355. doi: 10.3389/fcimb.2022.954355 (PMC9307962; doi:10.3389/fcimb.2022.954355)
Supplement: Supplementary file 5 [file Table_1.docx]

**Supplementary Table 1. Genome sequences and gene locus information of the analyzed strains.**

| **Species and strain names** | **Genome assembly No.** | **Biosample No.** | **Total sequence length** | **16S rRNA locus ID** | ***rpoB* locus ID** | ***gyrA***  **locus ID** | ***dnaA* locus ID** | ***recA* locus ID** | ***rplE* locus ID** | ***groL* locus ID** |
| --- | --- | --- | --- | --- | --- | --- | --- | --- | --- | --- |
| 1. *septimus (SAHP1)* | GCF_023614525.1 | SAMN28626158 | 1,917,962 | M9426_01465 | M9426_01920 | M9426_04560 | M9426_04585 | M9426_00275 | M9426_05865 | M9426_05960 |
| *P. vagnialis (KhD-2)* | GCF_900176605.1 | [SAMEA103970042](https://www.ncbi.nlm.nih.gov/biosample/SAMEA103970042/) | 1,877,211 | CAN42_RS00760 | CAN42_RS08325 | CAN42_RS02700 | CAN42_RS02725 | CAN42_RS06875 | CAN42_RS03895 | CAN42_RS03995 |
| *P. harei (NCTC13077)* | GCF_900638565.1 | SAMEA104224812 | 1,739,102 | EL281_RS01280 | EL281_RS02585 | EL281_RS00030 | EL281_RS00005 | EL281_RS04265 | EL281_RS06895 | EL281_RS06800 |
| *P. harei (NCTC13076)* | GCA_900454685.1 | [SAMEA4535753](https://www.ncbi.nlm.nih.gov/biosample/SAMEA4535753/) | 2,168,934 | NCTC13076_00613 | NCTC13076_02058 | NCTC13076_00865 | NCTC13076_00870 | NCTC13076_01648 | NCTC13076_01129 | NCTC13076_01147 |
| *P. harei (FDAARGOS1136)* | GCF_016726485.1 | [SAMN16357305](https://www.ncbi.nlm.nih.gov/biosample/SAMN16357305/) | 1,929,566 | I6I93_RS02785 | I6I93_RS01600 | I6I93_RS04800 | I6I93_RS04825 | I6I93_RS08645 | I6I93_RS06055 | I6I93_RS06150 |
| *P. phoceensis (SIT15)* | GCF_001517665.2 | [SAMEA3725799](https://www.ncbi.nlm.nih.gov/biosample/SAMEA3725799/) | 1,739,703 | BN3657_RS07960 | BN3657_RS05110 | BN3657_RS03245 | BN3657_RS03270 | BN3657_RS06570 | BN3657_RS00385 | BN3657_RS00290 |
| *P. timonensis (JC401)* | GCF_000312025.1 | [SAMEA2272625](https://www.ncbi.nlm.nih.gov/biosample/SAMEA2272625/) | 1,758,598 | HGPT_RS08625 | HGPT_RS00855***** | HGPT_RS06700 | HGPT_RS06675 | HGPT_RS02410 | HGPT_RS05195 | HGPT_RS05100 |
| *P. senegalensis (JC140)* | GCF_000321025.1 | [SAMEA2272315](https://www.ncbi.nlm.nih.gov/biosample/SAMEA2272315/) | 1,851,340 | PTSHG_RS08950 | PTSHG_RS03680 | PTSHG_RS07170 | PTSHG_RS07145 | PTSHG_RS01460 | PTSHG_RS05625 | PTSHG_RS05530 |
| *P. ovalis (MSJ-1)* | GCF_018919265.1 | [SAMN19373995](https://www.ncbi.nlm.nih.gov/biosample/SAMN19373995/) | 2,102,036 | KQI68_RS10225 | KQI68_RS07540 | KQI68_RS01335 | KQI68_RS01310 | KQI68_RS04305 | KQI68_RS03460 | KQI68_RS02360 |
| *P. lacydonensis (EL1)* | GCF_900106515.1 | [SAMEA4521260](https://www.ncbi.nlm.nih.gov/biosample/SAMEA4521260/) | 1,845,182 | BQ5450_RS03890 | BQ5450_RS03685 | BQ5450_RS05995 | BQ5450_RS06020 | BQ5450_RS00455 | BQ5450_RS08150 | BQ5450_RS07590 |
| *P. raoultii (KHD4)* | GCF_900099555.1 | [SAMEA4492522](https://www.ncbi.nlm.nih.gov/biosample/SAMEA4492522/) | 1,623,601 | BQ4446_RS03205 | BQ4446_RS07965 | BQ4446_RS01800 | BQ4446_RS01485 | BQ4446_RS05600 | BQ4446_RS07645 | BQ4446_RS01640 |
| *P. lacrimalis (NCTC13149)* | GCF_900454715.1 | [SAMEA48406918](https://www.ncbi.nlm.nih.gov/biosample/SAMEA48406918/) | 1,883,562 | DYH74_RS02265 | DYH74_RS02400 | DYH74_RS00030 | DYH74_RS00005 | DYH74_RS04690 | DYH74_RS03250 | DYH74_RS00235 |
| *P. mikwangii (ChDCB134)* | GCF_000468535.1 | [SAMN02469576](https://www.ncbi.nlm.nih.gov/biosample/SAMN02469576/) | 1,499,757 | M915_RS02395 | M915_RS03590 | M915_RS06360 | M915_RS06385 | M915_RS01645 | M915_RS04850 | M915_RS04115 |
| *P. asaccharolyticus (FDAARGOS1135)* | GCF_016791605.1 | [SAMN16357304](https://www.ncbi.nlm.nih.gov/biosample/SAMN16357304/) | 2,320,207 | I6I92_RS01495 | I6I92_RS00810 | I6I92_RS10630 | I6I92_RS10655 | I6I92_RS04460 | I6I92_RS01190 | I6I92_RS00310 |
| *P. asaccharolyticus (DSM 20463)* | GCF_900176115.1 | [SAMN00017477](https://www.ncbi.nlm.nih.gov/biosample/SAMN00017477/) | 2,232,586 | B8869_RS05485 | B8869_RS10675 | B8869_RS06625 | B8869_RS06650 | B8869_RS03030 | B8869_RS11080 | B8869_RS10175 |
| *P. stercorisuis (DSM 27563)* | GCF_017874395.1 | [SAMN18247114](https://www.ncbi.nlm.nih.gov/biosample/SAMN18247114/) | 1,795,400 | J2Z71_RS08875 | J2Z71_RS02015 | J2Z71_RS05475 | J2Z71_RS05450 | J2Z71_RS05220 | J2Z71_RS02215 | J2Z71_RS00465 |
| *P. obesi (ph1)* | GCF_000311865.1 | [SAMEA2272531](https://www.ncbi.nlm.nih.gov/biosample/SAMEA2272531/) | 1,773,998 | HGPB_RS08390 | HGPB_RS08025 | HGPB_RS03375 | HGPB_RS03400 | HGPB_RS04085 | HGPB_RS06615 | HGPB_RS02180 |
| *P. indolicus (NCTC11088)* | GCF_900454665.1 | [SAMEA4521465](https://www.ncbi.nlm.nih.gov/biosample/SAMEA4521465/) | 2,247,968 | DYE58_RS00870 | DYE58_RS02685 | DYE58_RS06720 | DYE58_RS06745 | DYE58_RS09370 | DYE58_RS04835 | DYE58_RS05345 |
| *P. nemausensis (1804121828)* | GCA_903819165.1 | [SAMEA6943338](https://www.ncbi.nlm.nih.gov/biosample/SAMEA6943338/) | 1,805,580 | PEPNEM18_00001 | PEPTYR26121_RS04680 | PEPTYR26121_RS03790 | PEPNEM18_00745 | PEPNEM18_00394 | PEPTYR26121_RS06930 | PEPTYR26121_RS04645 |
| *P. pacaensis (Kh-D5)* | GCF_900088125.1 | [SAMEA3996551](https://www.ncbi.nlm.nih.gov/biosample/SAMEA3996551/) | 1,851,572 | BN7444_RS00030 | BN7444_RS06190 | BN7444_RS04005 | BN7444_RS04030 | BN7444_RS08220 | BN7444_RS05710 | BN7444_RS03260 |
| *Anaerococcus degeneri (FDAARGOS1538)* | GCF_020215685.1 | [SAMN21460642](https://www.ncbi.nlm.nih.gov/biosample/SAMN21460642/) | 2,168,298 | LDJ82_RS02495 | LDJ82_RS08160 | LDJ82_RS05155 | LDJ82_RS05130 | LDJ82_RS01975 | LDJ82_RS03695 | LDJ82_RS07130 |

* One base in the gene was deleted to adapt the correct codon frame.
