# Supplementary material for: Identification of Peptoniphilus vaginalis-Like Bacteria, Peptoniphilus septimus sp. nov., From Blood Cultures in a Cervical Cancer Patient Receiving Chemotherapy: Case and Implications
Source: Front Cell Infect Microbiol. 2022 Jul 8;12:954355. doi: 10.3389/fcimb.2022.954355 (PMC9307962; doi:10.3389/fcimb.2022.954355)
Supplement: Supplementary file 6 [file Table_2.docx]

**Supplementary Table 2. 16S rRNA gene sequence identity of SAHP1 against other reference strains.**

| **Strain** | **Length of full 16S rRNA gene (bp)** | **% identity** |
| --- | --- | --- |
| *SAHP1* | 1525 | 100 |
| *P. vaginalis (KhD-2)* | 1525 | 99.02 |
| *P. harei (NCTC13077)* | 1525 | 98.69 |
| *P. harei (NCTC13076)* | 1525 | 98.65 |
| *P. harei (FDAARGOS1136)* | 1525 | 98.65 |
| *P. phoceensis (SIT15)* | 1525 | 97.25 |
| *P. timonensis (JC401)* | 1525 | 96.98 |
| *P. senegalensis (JC140)* | 1524 | 96.13 |
| *P. ovalis (MSJ-1)* | 1525 | 96.07 |
| *P. lacydonensis (EL1)* | 1525 | 95.67 |
| *P. raoultii (KHD4)* | 1526 | 92.27 |
| *P. lacrimalis (NCTC13149)* | 1527 | 92.14 |
| *P. mikwangii (chDCB134)* | 1529 | 89.21 |
| *P. asaccharolyticus (DSM20463)* | 1529 | 88.75 |
| *P. asaccharolyticus (FDARRGOS1135)* | 1529 | 88.75 |
| *P. stercorisuis (DSM27563)* | 1530 | 88.56 |
| *P. obesi (ph1)* | 1528 | 88.49 |
| *P. indolicus (NCTC11088)* | 1529 | 87.97 |
| *P. nemausensis (1804121828)* | 1528 | 87.25 |
| *P. pacaensis (Kh-D5)* | 1528 | 86.80 |
| *Anaerococcus degeneri (FDAARGOS1538)* | 1528 | 85.31 |

The identities were calculated by DNAMAN software.
