# Supplementary material for: Identification of Peptoniphilus vaginalis-Like Bacteria, Peptoniphilus septimus sp. nov., From Blood Cultures in a Cervical Cancer Patient Receiving Chemotherapy: Case and Implications
Source: Front Cell Infect Microbiol. 2022 Jul 8;12:954355. doi: 10.3389/fcimb.2022.954355 (PMC9307962; doi:10.3389/fcimb.2022.954355)
Supplement: Supplementary file 8 [file Table_4.docx]

**Supplementary Table 4. AAI between SAHP1 and other reference strains.**

| #Genome A | Genes in A* | Genome B | Genes in B | orthologous genes# | Mean AAI (%) | Std. AAI (%)^ | Orthologous fraction (OF, %)! |
| --- | --- | --- | --- | --- | --- | --- | --- |
| SAHP1 | 1805 | *P. vaginalis (KhD-2)* | 1748 | 1405 | 93.51 | 7.72 | 80.38 |
| SAHP1 | 1805 | *P.harei (NCTC13076)* | 2149 | 1384 | 91.33 | 8.14 | 76.68 |
| SAHP1 | 1805 | *P.harei (NCTC13077)* | 1586 | 1352 | 91.31 | 7.94 | 85.25 |
| SAHP1 | 1805 | *P.harei (FDAARGOS1136)* | 1786 | 1364 | 91.30 | 8.13 | 76.37 |
| SAHP1 | 1805 | *P. phoceensis (SIT15)* | 1615 | 1294 | 86.78 | 9.9 | 80.12 |
| SAHP1 | 1805 | *P. timonensis (JC401)* | 1874 | 1107 | 86.72 | 10.13 | 61.33 |
| SAHP1 | 1805 | *P. senegalensis (JC140)* | 1724 | 1295 | 82.8 | 12.12 | 75.12 |
| SAHP1 | 1805 | *P. lacydonensis (EL1)* | 1747 | 1276 | 80.34 | 12.3 | 73.04 |
| SAHP1 | 1805 | *P. ovalis (MSJ-1)* | 2008 | 1250 | 74.71 | 13.15 | 69.25 |
| SAHP1 | 1805 | *P lacrimalis (NCTC13149)* | 1761 | 1131 | 65.46 | 15.47 | 64.22 |
| SAHP1 | 1805 | *P. raoultii (KHD4)* | 1591 | 1064 | 65.03 | 15.01 | 66.88 |
| SAHP1 | 1805 | *P. obesi (ph1)* | 1669 | 1052 | 63.11 | 17.19 | 63.03 |
| SAHP1 | 1805 | *P. asaccharolyticus (DSM20463)* | 2284 | 1073 | 60.43 | 15.23 | 59.45 |
| SAHP1 | 1805 | *P. asaccharolyticus (FDAARGOS1135)* | 2328 | 1104 | 60.28 | 15.21 | 61.16 |
| SAHP1 | 1805 | *P. mikwangii (ChDCB134)* | 1398 | 921 | 59.82 | 14.06 | 65.88 |
| SAHP1 | 1805 | *P. stercorisuis (DSM 27563)* | 1715 | 1000 | 59.21 | 13.54 | 58.31 |
| SAHP1 | 1805 | *P. indolicus (NCTC11088)* | 2152 | 1101 | 59.15 | 14.37 | 61.00 |
| SAHP1 | 1805 | *P. nemausensis (1804121828)* | 1709 | 905 | 57.58 | 16.96 | 52.95 |
| SAHP1 | 1805 | *P. pacaensis (KhD5)* | 1747 | 908 | 55.56 | 14.69 | 51.97 |
| SAHP1 | 1805 | *Anaerococcus degeneri (FDAARGOS1538)* | 1987 | 731 | 53.52 | 14.78 | 40.5 |
| SAHP1 | 1805 | *P. gorbachii (DSM 21461)* | 1788 | 1379 | 83.53 | 11.66 | 77.13 |
| SAHP1 | 1805 | *P. grossensis (ph5)* | 1985 | 1424 | 83.33 | 12.28 | 78.89 |

*The protein-coding gene number of the genome was obtained by using the compareM packages and was tinily different with those by annotated in COG, KEGG, Go, NCBI Reference, RefSeqPfam, and TIGRFAMs databases (1805 vs. 1804) .

# Due to different algorithms, there are small differences between the orthologous genes numbers obtained from compareM and OrthoVenn2.

^Std. indicates standard deviation.

!The orthologous fraction (OF) between the two genomes, defined as the number of orthologs genes divided the minimum number of genes in either genome

Cells filled gray indicate that the data was obtained from the genome sequence of inclusive taxonomy checked strain.
