# Supplementary material for: Identification of Peptoniphilus vaginalis-Like Bacteria, Peptoniphilus septimus sp. nov., From Blood Cultures in a Cervical Cancer Patient Receiving Chemotherapy: Case and Implications
Source: Front Cell Infect Microbiol. 2022 Jul 8;12:954355. doi: 10.3389/fcimb.2022.954355 (PMC9307962; doi:10.3389/fcimb.2022.954355)
Supplement: Supplementary file 9 [file Table_5.docx]

**Supplementary Table 5. Number of protein-coding genes annotated by different database for SAHP1.**

| **Database** | **Annotated number** | **% all** |
| --- | --- | --- |
| **COG** | 1394 | 77.27 |
| **KEGG** | 960 | 53.22 |
| **GO** | 1075 | 59.59 |
| **Refseq** | 1774 | 98.34 |
| **Pfam** | 1517 | 84.09 |
| **TIGRFAMs** | 612 | 33.92 |
| **all databases** | 479 | 26.55 |
| **at least one databases** | 1778 | 98.56 |
| **Overall** | 1804 | 100 |
