# Supplementary material for: Identification of Peptoniphilus vaginalis-Like Bacteria, Peptoniphilus septimus sp. nov., From Blood Cultures in a Cervical Cancer Patient Receiving Chemotherapy: Case and Implications
Source: Front Cell Infect Microbiol. 2022 Jul 8;12:954355. doi: 10.3389/fcimb.2022.954355 (PMC9307962; doi:10.3389/fcimb.2022.954355)
Supplement: Supplementary file 10 [file Table_6.docx]

**Supplementary Table 6. Details of antibiotic-resistance genes of SAHP1 annotated by the CARD database.**

| Locus ID | sseqid | dbxref | Gene name | Start and end | coverage | Identity (%) |
| --- | --- | --- | --- | --- | --- | --- |
| M9426_07000 | AM990992.1:1003680-1001760 | card:AM990992.1:1003680-1001760 | tetM | 1-1920/1920 | 100 | 99.27 |
| M9426_08580 | X03216.1:5282-4550 | card:X03216.1:5282-4550 | ErmA | 1-732/732 | 100 | 82.51 |
| M9426_08985 | AY712687:0-831 | card:AY712687:0-831 | aad(6) | 1-831/831 | 100 | 100 |
| M9426_08990 | U01945:373-916 | card:U01945:373-916 | SAT-4 | 1-543/543 | 100 | 99.82 |
| M9426_08995 | CP004067:52914-53709 | card:CP004067:52914-53709 | APH(3')-IIIa | 1-795/795 | 100 | 99.87 |

sseqid, subject or target (e.g., reference genome) sequence id; dbxref, database cross-reference: pointer to related information in another database.
